# Optimizing non-Newtonian fluids for impact protection of laminates

**DOI:** 10.1073/pnas.2317832121

**Published:** 2024-02-27

**Authors:** James A. Richards, Daniel J. M. Hodgson, Rory E. O’Neill, Michael E. DeRosa, Wilson C. K. Poon

**Affiliations:** ^a^Edinburgh Complex Fluids Partnership, School of Physics and Astronomy, The University of Edinburgh, James Clerk Maxwell Building, Peter Guthrie Tait Road, Edinburgh EH9 3FD, United Kingdom; ^b^Science and Technology Division, Corning Incorporated, Corning, NY 14831

**Keywords:** shear thickening, fluid–solid interaction, shear thinning, smart materials

## Abstract

Non-Newtonian fluids can be used for the protection of flexible laminates. Understanding the coupling between the flow of the protecting fluid and the deformation of the protected solids is necessary in order to optimize this functionality. We present a scaling analysis of the problem based on a single coupling variable, the effective width of a squeeze flow between flat rigid plates, and predict that impact protection for laminates is optimized by using shear-thinning, and not shear-thickening, fluids. The prediction is verified experimentally by measuring the velocity and pressure in impact experiments. Our scaling analysis should be generically applicable for non-Newtonian fluid–solid interactions in diverse applications.

## Significance

Complex fluids that alter their mechanical response as the applied forces change enable smart materials. A prime example is flexible body armor infused with a shear-thickening suspension that hardens on impact. During impact, there is a complex interplay between solid deformation and fluid flow that complicates predictive design. We construct and experimentally validate a theoretical model for a fluid–solid laminate that describes display glass applications, such as in smartphones. Strikingly, we find that, now, sandwiching a fluid that becomes less viscous during impact between a top and a bottom layer protects both against impact. Our approach establishes design principles for smart fluid–solid composites.

Woven fabrics impregnated with a shear-thickening colloidal fluid, whose viscosity increases suddenly at a critical shear rate, can function as body armor (1). Perhaps surprisingly, the shear-thickening fluid does not directly provide protection in body armor because of the bulk rheology that allows, for example “running on cornstarch” (2) due to propagating jamming fronts (3). Instead, as the fibers are pulled past one another the suspension between them jams, preventing them from being pulled apart and increasing effective inter-fiber friction (4), so that they form a rigid layer to spread impact and protect the material underneath.

Partly inspired by this application, there is growing interest in smart materials that incorporate various non-Newtonian fluids in solid structures (5–9). In particular, in direct analogy with body armors, it is envisaged that including shear-thickening fluids in laminates may provide impact protection. However, analyzing the impact response of fluid–solid composites is challenging even in the case of Newtonian fluids (10). Deformation of the solid drives fluid flow, which then generates a pressure, which in turn changes the solid deformation, creating feedback. For a non-Newtonian fluid, such fluid–solid interaction is even more challenging, because the fluid property changes as the flow develops throughout impact, and analyses to date are limited, e.g., to blood flow (11–13), or stationary process such as blade coating (14).

We consider fluid–solid interactions in a laminate consisting of a non-Newtonian fluid sandwiched between a flexible sheet above and a rigid base below, which is a model for various real-life applications, e.g., a display in which the base layer is an LCD panel and the top layer is a piece of glass, both of which must be protected from concentrated impacts at $≲O(10\;{\text{ms}}^{-1})$. The physics differs from that in shear thickening body armor. The requirement here is to protect both solid layers, while body armor is optimized for the protection of the single lower layer.

We perform a scaling analysis of the coupling between fluid flow, rheology, and solid deformation in our geometry based on the idea of an “effective squeeze flow width,” and verify our analysis using controlled-velocity impact experiments. We find that the effective squeeze flow width varies weakly throughout the impact, so that the process can be approximated as a simple rigid squeeze flow. From this, we find, surprisingly, that shear thinning, not thickening, is optimal for protection.

## Results

### Modeling.

**Fig. 1.**
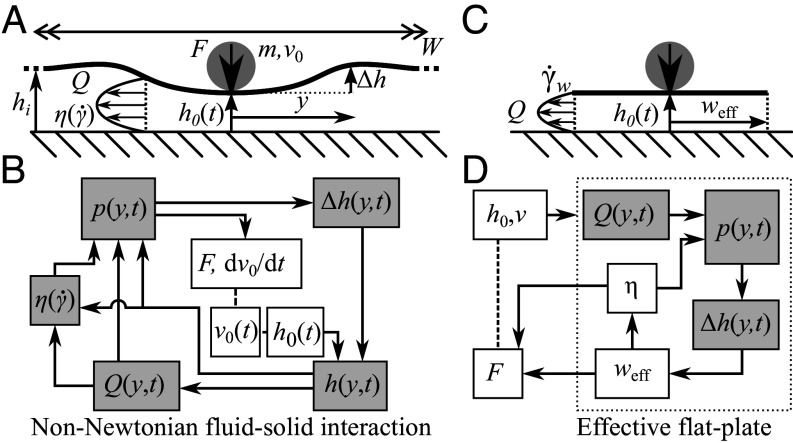
Non-Newtonian fluid–solid interaction. (*A*) Diagram of point impact on simplified laminate geometry. (*B*) Schematic of full coupling between fluid rheology, fluid flow, and glass deformation. (*C*) Diagram of simplified effective plate. (*D*) Schematic of simplified closure with single effective plate width variable.

Using a quasi-2D setup, we analyze the downward impact of a point mass *m* at the origin, y=0, with speed *v* on a flexible plate initially at height $h_{i}$ parallel to a rigid bottom plate, with the gap filled by a fluid, Fig. 1*A*. The width of the plate $W\gg h_{i}$, and breadth of the plate (perpendicular to the page) $L\gg h_{i}$. The upper plate is pushed down, leaving a gap $h_{0}\left(t\right)$ at the impact point, and bending deformation Δh(y,t) upward. The net motion causes a fluid flow, *Q*. If the impact velocity is significantly sub-sonic, i.e., $v_{0}\ll O\;\left(1,000\;{\text{ms}}^{-1}\right)$ for most solids and liquids, then incompressibility and mass conservation require:

[1] $$\begin{array}{c} \frac{\partial }{\partial t}\left[h_{0}\left(t\right)+\Delta h\left(y,t\right)\right]=-\frac{\partial Q}{\partial y}, \end{array}$$

with *y* the distance from the impact. The pressure gradient associated with the impact-driven flow is given by:

[2] $$\begin{array}{c} \frac{dp}{dy}=-\frac{12\eta Q}{h^{3}}, \end{array}$$

where the fluid viscosity *η* is constant for a Newtonian fluid. The pressure, p(y,t), which satisfies p(y→∞)=0, pushes back on the impacting mass *m*,

[3] $$\begin{array}{c} m\frac{dv}{dt}=m\frac{d^{2}h_{0}\left(t\right)}{dt^{2}}=-L\int_{\infty }^{+\infty }dyp\left(y,t\right), \end{array}$$

and bends the flexible layer, which has thickness $h_{g}$ and rigidity $B=ELh_{g}^{3}/12$ (with *E* its Young’s modulus). The shape of the layer follows the Euler-Bernoulli equation (15),

[4] $$\begin{array}{c} \frac{B}{L}\frac{\partial^{4}\Delta h}{\partial y^{4}}=p\left(y,t\right), \end{array}$$

where we have neglected the laminate mass as ≪m. Self-consistency requires that Eq. **4** solves to give small plate deflection, so that flow is essentially along *y*, as is assumed in the “lubrication approximation” (16), Eq. **2**.

The coupled integro-differential equations, Eqs. **1** to **3**, need to be supplemented by a form for the rate-dependent viscosity, $\eta (\dot{\gamma })$, if the fluid is non-Newtonian. The complex feedback between quantities, Fig. 1*B*, means that finite element or immersed boundary numerical methods are needed to solve specific fluid–solid interaction problems for Newtonian (10) and non-Newtonian fluids (17, 18); but such solutions offer little physical insight into fluid–solid interactions, for which we turn to a different approach.

#### Simplified closure.

To analyze the fluid–solid interactions in our geometry, note first that since the pressure gradient $\partial_{y}p\propto h^{-3}$, we need only consider the region around the impact where deformation is small, $\Delta h≲h_{0}$.^*^ Within this region the surface is only weakly curved, and a calculation of the shear rate shows that it is adequate to treat it as a flat surface, $h\left(y\right)\approx h_{0}$ (*SI Appendix*, Fig. S1). We therefore define an effective flat plate width, $w_{\mathrm{eff}}$, such that the pressure created by a rigid plate squeeze flow bends the flexible plate by $\Delta h=h_{0}$ at $y=w_{\mathrm{eff}}$. The squeeze flow for $\left|y\right|\leq w_{\mathrm{eff}}\ll W$ is solved analytically (19), but we neglect fluid flow and deformation outside ($|y|>w_{\mathrm{eff}}$), Fig. 1 *C* and *D*. Within this local approximation, boundary conditions can be neglected as volume conservation will be ensured by, e.g., the surface being pushed up further away from the impact zone.

We use a scaling analysis to determine $w_{\mathrm{eff}}$, which is not known a priori. The flux created by the rigid-plate squeeze flow $Q≃vw_{\mathrm{eff}}$ gives $\partial_{y}p≃12\eta vw_{\mathrm{eff}}/h_{0}^{3}$ and $p≃12w_{\mathrm{eff}}^{2}\eta v/h_{0}^{3}$. Eq. **4** implies that the deflection $\Delta h≃pw_{\mathrm{eff}}^{4}\times L/B$. Self consistency demands that this $\Delta h\approx h_{0}$, which combines with *p* to give:

[5] $$\begin{array}{c} w_{\mathrm{eff}}≃{\left(\frac{Bh_{0}^{4}}{12\eta vL}\right)}^{\frac{1}{6}},\frac{F}{L}≃pw_{\mathrm{eff}}≃\frac{12\eta vw_{\mathrm{eff}}^{3}}{h_{0}^{3}}={\left(\frac{12\eta vB}{Lh_{0}^{2}}\right)}^{\frac{1}{2}}. \end{array}$$

While higher *η*, faster *v*, and narrower $h_{0}$ bend the plate more strongly and reduce $w_{\mathrm{eff}}$, the dependence is weak. The somewhat unusual $\frac{1}{6}$ exponent is traceable to the dependence of plate deflection on $w_{\mathrm{eff}}^{6}$.^†^ The nearly-constant $w_{\mathrm{eff}}$ means that the dynamics can be thought of as a modified fixed width squeeze flow that scales approximately as $h_{0}^{-3}$.

To capture the lowest order effects of a rate-dependent viscosity, $\eta =\eta (\dot{\gamma })$, in non-Newtonian fluids, a further approximation is made. We take the fluid to be effectively Newtonian with a single viscosity, $\eta_{\mathrm{eff}}=\eta \left({\dot{\gamma }}_{w}\right)$, where ${\dot{\gamma }}_{w}$ is the shear rate at the edge of the effective plate ($y=w_{\mathrm{eff}}$) for a fluid of this viscosity. This again ensures self-consistency; it also recalls the use of the rim shear rate in calculating the viscosity in parallel-plate rheometry (20).

We use a power-law model, $\eta_{\mathrm{eff}}=K{\dot{\gamma }}_{w}^{n-1}$, to explore the effect of thinning (n<1) and thickening (n>1) on impact protection. Now, Eq. **5** becomes (*SI Appendix*)

[6] $$\begin{array}{c} \begin{array}{c} w_{\mathrm{eff}}\propto {\left(\frac{Bh_{0}^{4}}{12KvL}\right)}^{\frac{1}{6}}{\left(\frac{{\left(6v\right)}^{5}B}{2h_{0}^{8}KL}\right)}^{\frac{1-n}{6(n+5)}}\\ \mathrm{and}\;\frac{F}{L}\propto \frac{\sqrt{12KvB/L}}{h_{0}}{\left(\frac{{\left(6v\right)}^{5}B}{2h_{0}^{8}KL}\right)}^{\frac{n-1}{2(n+5)}}, \end{array} \end{array}$$

which reduce to Newtonian results, Eq. **5**, for K=η and n=1. Eq. **6** gives the force per unit length in terms of (B/L,K,n) and a single dynamical variable $h_{0}\left(t\right)$ with its derivative ${\dot{h}}_{0}=v$; this then allows us to understand how a flexible solid-fluid laminate may be protected against impact.

#### Numerical solutions.

After impact, a time-dependent bending moment $M\left(t\right)=F\left(t\right)w_{\mathrm{eff}}\left(t\right)$ develops, which flexes the upper plate, Eq. **4**. Large flexure can lead to breakage when *M* exceeds a critical bending moment, $M^{*}$. Protection requires minimizing the maximum, $M_{\max}<M^{*}$, e.g., for a given geometry through fluid optimization.

A Newtonian-fluid laminate with initial gap $h_{i}$ impacted by mass *m* at initial downward speed $v_{i}$ obeys from Eq. **3**

[7] $$\begin{array}{c} \frac{d^{2}h_{0}}{dt^{2}}=-\frac{C}{h_{0}}{\left|\frac{dh_{0}}{dt}\right|}^{\frac{1}{2}},\;C=\sqrt{\frac{12\eta BL}{m^{2}v_{i}^{3}}}. \end{array}$$

**Fig. 2.**
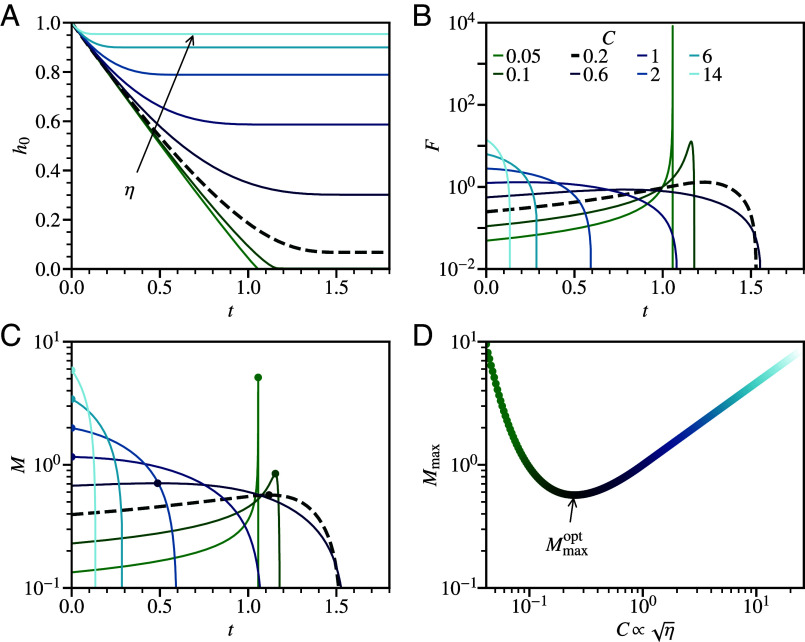
Predicted response to impact for a Newtonian fluid laminate with varying viscosity. (*A*) Changing gap, $h_{0}\left(t\right)$, normalizing length $h_{i}$ and time $h_{i}/v_{i}$. Lines: green to light blue with increasing viscosity, *η*, setting impact parameter, $C={\left(12\eta BL\right)}^{0.5}/mv_{i}^{3/2}$, see legend in (*B*). Bold dashed line for C=0.2 at optimum viscosity; see (*D*). (*B*) Impact force, *F*, normalized by ${\left(L\eta v_{i}B\right)}^{0.5}/h_{i}$. (*C*) Bending moment, $M\left(t\right)=Fw_{\mathrm{eff}}$, with effective plate width $w_{\mathrm{eff}}$ normalized by ${\left(Bh_{i}^{4}/Lv_{i}\eta \right)}^{1/6}$ and peak $M\left(t\right)=M_{\max}$ (circle). (*D*) Peak bending moment, $M_{\max}$ vs *C*.

The gap and time have been normalized by $h_{i}$ and $h_{i}/v_{i}$, giving a single dimensionless “impact parameter,” *C*, which captures the ratio of viscous dissipation, $F\left(h_{i}\right)\times h_{i}\propto \sqrt{v_{i}}$, to kinetic energy, $\propto v_{i}^{2}$. We solve for $h_{0}\left(t\right)$ numerically (using SciPy v1.10.1 integrate.odeint) for various $C\propto \sqrt{\eta }$, Fig. 2.

At large *η* (C=14, 6, 2), the impact is rapidly stopped and the gap hardly drops, $h_{0}\left(t\to \infty \right)≲1$, Fig. 2*A* [light blue lines, see legend in Fig. 2*B*]. This causes a large initial force, F(0), Fig. 2*B*, and maximum bending moment $M_{\max}=M\left(0\right)$ that grows with *η*, Fig. 2*C* (circle); however, both F(t) and M(t) drop rapidly. At intermediate *η* (C=1, 0.6), $h_{0}$ decreases noticeably before stabilizing, while F(0) and M(0) both drop, but F(t) and M(t) stay constant for longer before dropping rapidly. At the smallest *η* (C=0.1,0.05), the impact is not slowed and $h_{0}\to 0$, giving a sharp peak in F(t), Fig. 2*B*, and in M(t) (as $w_{\mathrm{eff}}$ changes sub-linearly with $h_{0}$) that now grows as η→0, Fig. 2*C*.

At some optimal C≈0.2, $M_{\max}$ is minimized at $M_{\max}^{\mathrm{opt}}$, Fig. 2*D*. The impact is absorbed over the whole gap with a near-constant $v={\dot{h}}_{0}$, but eventually slows before *F* diverges. As $w_{\mathrm{eff}}$ is weakly dependent on $h_{0}$, reducing the divergence in *F* directly gives a flatter M(t). This, however, still peaks as the gap narrows, Fig. 2*C* [bold dashed line], increasing 50% from t=0 before dropping rapidly to zero. To obtain a minimum $M_{\max}$ with a flat M(t) profile, we turn to non-Newtonian fluids.

**Fig. 3.**
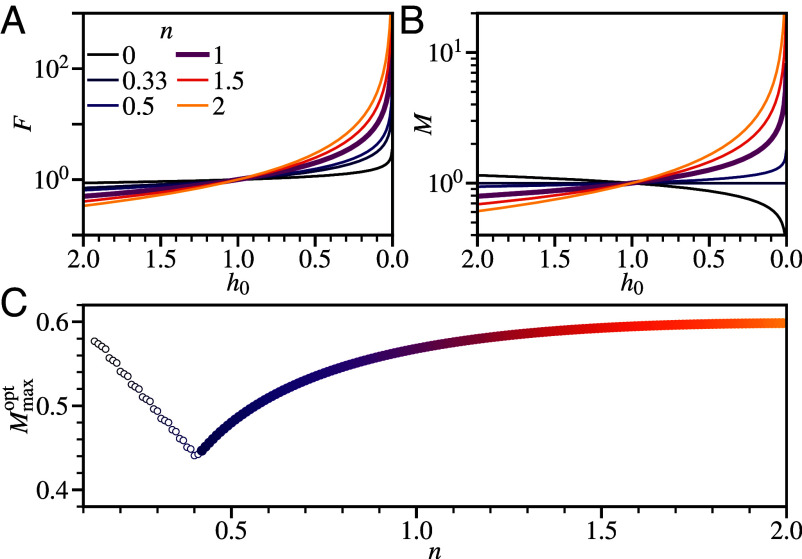
Predicted impact response for a power-law fluid. (*A*) Constant velocity impact force, $F(h_{0})$, at different index, *n*, dark (purple) thinning to light (yellow) shear-thickening (*Legend*). Normalized by setting additional parameters to unity. (*B*) Corresponding bending moment, $M(h_{0})$. (*C*) Peak M(t) for decelerating impact vs power-law index, $M_{\max}^{\mathrm{opt}}\left(n\right)$, at optimal consistency, *K*, following Fig. 2*D*. Symbols: light (thickening) to dark (thinning); open, impact to $h_{0}\left(t\right)<10^{-4}$.

Consider first a constant-speed impact. We plot in Fig. 3*A* and *B* the $h_{0}\left(t\right)$ dependence implied by Eq. **6**:

[8] $$\begin{array}{c} F\propto h_{0}^{-\frac{5n+1}{n+5}}\;\text{and}\;M=Fw_{\mathrm{eff}}\propto h_{0}^{-\frac{3n-1}{n+5}}. \end{array}$$

The force and bending moment in a shear-thickening fluid laminate (n=1.5, 2) diverge more sharply as the gap narrows than the Newtonian case (n=1). However, a shear-thinning fluid (n=0.5,0.33,0) leads to a weaker force divergence. For n=0.5, the bending moment also diverges more weakly than the Newtonian case. Interestingly, decreasing *n* further brings a constant *M* (n=0.33) and then a decreasing *M* (n=0). These results suggest that for laminate protection, a shear-thinning, not thickening, fluid is needed.

We next confirm and generalize our analysis with numerical solutions of the dynamical equation for $h_{0}\left(t\right)$:

[9] $$\begin{array}{c} \frac{d^{2}h_{0}}{dt^{2}}=-\frac{\sqrt{12Kv}}{h_{0}}{\left(\frac{{\left(6v\right)}^{5}}{2Kh_{0}^{8}}\right)}^{\frac{n-1}{2(n+5)}}, \end{array}$$

where the second term modifies the Newtonian equation, Eq. **7**, and *B*, *L*, and *m* have been set to unity.

**Fig. 4.**
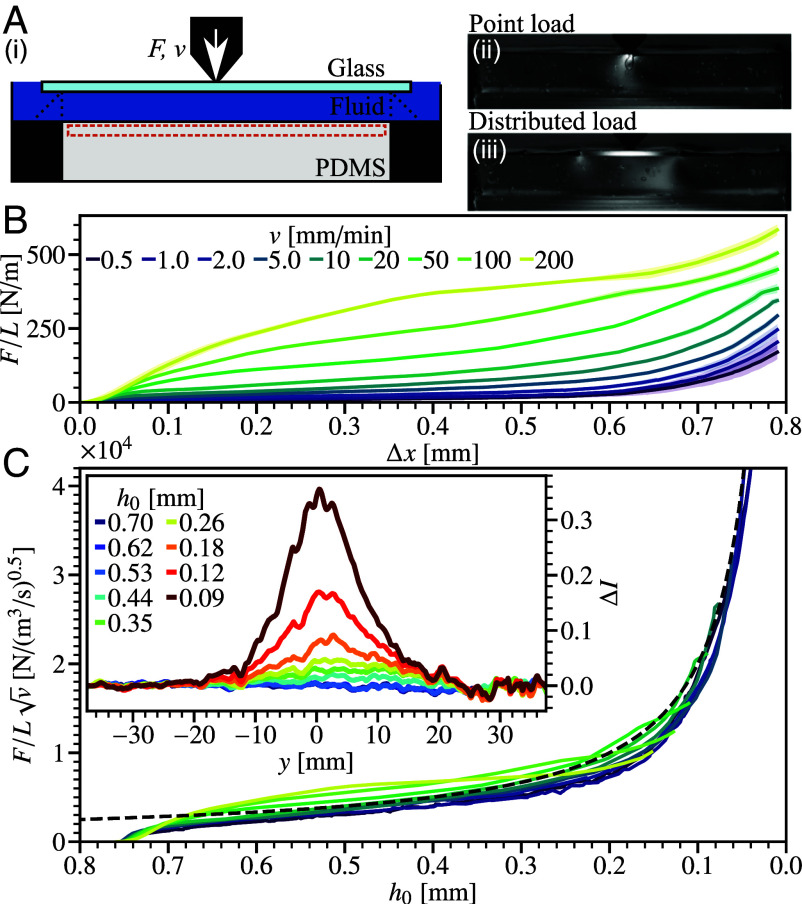
Experimental controlled-velocity impact into a Newtonian fluid laminate. (*A*) Testing apparatus. (*i*) Diagram from *Top* to *Bottom*: 0.3 mm glass; 0.70 to 0.76 mm fluid layer; base with 10 mm PDMS, region analyzed for pressure measurement, dashed (orange) outline. Fluid flow out of plane prevented by rigid glass panes (light shading); laterally serrated anvils allow fluid flow during loading, *SI Appendix*, Fig. S2. (*ii*) Image, I(x,y), of static point loading, $F/L\approx 200\;{\text{Nm}}^{-1}$, directly on PDMS in dark-field circular polariscope. Note, background subtraction has not been performed and the air pocket created is unique to the localized load directly on the PDMS. (*iii*) Distributed static load, across 20 mm rigid glass slide. (*B*) Force–displacement response with varying speed, *v*, for 0.3 mm thick glass with 0.76 mm initial gap. Lines: dark (purple) to light (yellow), slow to fast controlled *v* (*Inset* legend), three test average, SD shown by shading. (*C*) Velocity-normalized force, $F/L\sqrt{v}$, as a function of corrected gap, $h_{0}$, line shading as in (*B*). Dashed black line: model prediction, $F/L=12{\left(\eta vB/L\right)}^{1/2}/h_{0}$. *Inset*: polariscope proxy pressure measurement. Vertically averaged intensity change, ΔI(y), across quasi-2D geometry at decreasing $h_{0}$ [blue to dark (red), see *inset* legend]. Impact velocity, v= 20 mm^−1^ and $h_{i}=$ 0.7 mm.

For any value of n≳0.4, we find an optimal *K* for which the maximum bending moment is minimized (comparable to Fig. 2*D*, but with η→K). Increasing *n* from the Newtonian value of unity, this optimal value $M_{\max}^{\mathrm{opt}}$ increases, Fig. 4*C*, i.e., a shear-thickening fluid decreases protection. In contrast, decreasing *n* below unity, i.e., changing to progressively more shear-thinning fluids, lowers $M_{\max}^{\mathrm{opt}}$, thus offering increasing impact protection, consistent with our constant-*v* analysis.

For n<0.4, we find that decreasing *K* below its optimal value brings laminate failure, as $h_{0}\to 0$. So, we predict that optimal impact protection is offered by a shear-thinning fluid with n=0.4, somewhat higher than the $\frac{1}{3}$ from the constant-*v* analysis, but is insensitive to pre-factors in our scaling analysis. Physically, a shear-thinning fluid is optimal as it is harder to push out of large gaps (low $\dot{\gamma }$, higher $\eta_{\mathrm{eff}}$, larger *F*) than for narrow gaps (high $\dot{\gamma }$, lower $\eta_{\mathrm{eff}}$, smaller *F*), which smooths F(t) and hence M(t).

### Constant Velocity Experiments.

We verify our analysis in an experimental realization of our quasi-2D setup from Fig. 1*A*, using a universal testing machine to drive a wedge downward at a laminate consisting of a fluid sandwiched between a 0.3-mm-thick flexible glass plate and a 10-mm-thick polydimethylsiloxane (PDMS) base, Fig. 4*A*, at low enough constant velocity, *v*, to allow us to follow the force on the wedge, *F*, as a function of time, or, equivalently, (downward) displacement, Δx. The gap height is $h_{0}=h_{i}-\Delta x+F/k$, where $h_{i}$ is the initial gap height, and *k* is the (separately measured) stiffness of the system. We measured F(Δx) at different imposed *v*, and monitored the pressure on the PDMS via photoelastic imaging. Experimental details are in *Materials and Methods*.

#### Newtonian fluids.

We begin with a Newtonian fluid laminate with $h_{i}=$ 0.7 mm, using glycerol as the “sandwich filling,” increasing *v* from 0.5 mm min^−1^, Fig. 4*B* [dark (purple) lines], to 200 mm min^−1^ [light (yellow) lines]. At low *v*, the fluid can almost freely drain and *F* is low, only increasing as Δx→0.8mm and $h_{0}\to 0$. With increasing *v*, F(Δv) takes on a sigmoidal shape. Converting Δx to $h_{0}$ and normalizing by $\sqrt{v}$ collapses the data to within a factor of 1.5 over a 400-fold variation in *v*, Fig. *C*. Confirming the $\sqrt{v}$ scaling of Eq. **5**. Indeed, $F/L=12{\left(\eta vB/Lh_{0}^{2}\right)}^{1/2}$ offers a credible account of the collapsed data (dashed line). That this is within an order-unity numerical factor ($\sqrt{12}\approx 3.5$) of Eq. **5** validates the physics embodied in our scaling analysis: an effective squeeze flow that shrinks in extent as the viscous forces more strongly bend the flexible upper layer.

To illustrate this physics, we turn to photoelastic measurements, where light intensity is a proxy for the pressure, so that we can visually distinguish between a point and a distributed load, Fig. 4 *A*, *ii* and *iii*, respectively. At $v=20\;\text{mm}\;{\text{min}}^{-1}$, a bright region, evidencing high pressure, emerges at $h_{0}≲0.35\;\text{mm}$ Fig. 4*C*, and grows in intensity as $h_{0}$ decreases further. The half width of a Gaussian fitted to the measured intensity pattern decreases only weakly, from 9.9(2) to 6.19(3) mm as $h_{0}$ decreases from 0.53 to 0.09 mm. The observation of a localized high-pressure region is consistent with assumption of squeeze flow in a confined region of some effective width $w_{\mathrm{eff}}$. The weak dependence of $w_{\mathrm{eff}}$ on $h_{0}$ is also consistent with Eq. **5**, from which we predict $w_{\mathrm{eff}}≃{\left(Bh_{0}^{4}/12L\eta v\right)}^{1/6}=9\text{mm}$ at $h_{0}=0.35\;\text{mm}$ down to $w_{\mathrm{eff}}≃4\;\text{mm}$ at $h_{0}=0.09\;\text{mm}$, comparable to the observed widths and trends of the high-pressure region. Finally, these results are consistent with our assumptions of lubrication flow ($w_{\mathrm{eff}}\gg h_{0}$) and neglecting boundaries ($w_{\mathrm{eff}}\ll W=75\;\text{mm}$). Thus, the complex feedback between fluid flow and plate deformation can indeed be captured in an “effective flat plate” treatment.

#### Non-Newtonian fluids.

**Fig. 5.**
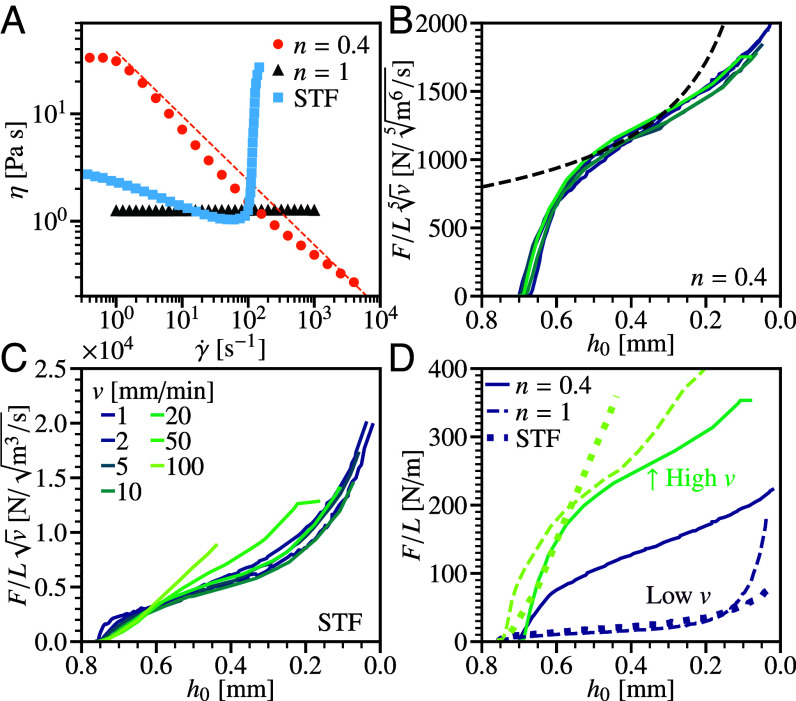
Impact into non-Newtonian fluid based laminates. (*A*) Fluid rheology, viscosity with shear rate, $\eta (\dot{\gamma })$. Symbols: light (blue) squares, shear-thickening suspension of 20 wt% fumed silica in PEG200 measured with fixed stress; (orange) circles, 7 wt% suspension of hydrophobic fumed silica in PEG200 measured at fixed rate; and dark (gray) triangles, glycerol. Dashed line, representative power-law fit for shear-thinning region, $\eta =K{\dot{\gamma }}^{n-1}$, for n=0.4, K=38 Pa s^0.4^ and $\dot{\gamma }=$ 1 to $10^{4}$ s^−1^. (*B*) Shear-thinning fluid. Force, $F(h_{0})/L$, normalized by speed, $\sqrt[{5}]{v}$, for $h_{i}=0.70\;\text{mm}$. Lines: solid, dark (purple) to light (green), v= 1 mm min^−1^ to 20 mm min^−1^, see legend in part (*B*); dashed, model prediction [Eq. **6**, n=0.4, 2.4 pre-factor]. (*C*) Shear-thickening fluid. Force normalized for Newtonian fluid by $\sqrt{v}$. Lines, increasing *v*, see *inset* legend. (*D*) Comparison of force response for different fluid rheologies. Dark lines, low speed, $v=1\;\text{mm}\;{\text{min}}^{-1}$: thin solid, shear thinning; dashed, Newtonian; and thick dotted, STF. Light lines, high *v*, v=20, 50 and 100 mm min^−1^ respectively.

We next tested a laminate filled with an n=0.4 shear-thinning suspension, Fig. 5*A* (filled circles); this and the shear-thickening suspension can be treated as continua, as the particle size is much smaller than the minimum gap (*SI Appendix*). Now, Eq. **6** predicts $F\propto v^{0.22}$, consistent with the observed collapse of $F(h_{0})$ data taken at different speeds when we plot $F\left(h_{0}\right)/\sqrt[{5}]{v}$, Fig. 5*B*. The prediction of $F\propto h_{0}^{-0.55}$ [Eq. **8**] does not capture the transient, early-stage response, but shows moderate agreement at intermediate $h_{0}$, Fig. 5*B* (dashed), with a prefactor of 2.4 consistent with a scaling analysis. The observed divergence in *F* as $h_{0}\to 0$ is weaker than for n=1, matching the predicted trend. However, it is also weaker than predicted for n=0.4. Better agreement between theory and experiment here may require more careful modeling of shear-thinning fluids under squeeze flow conditions (21).

If instead a shear-thickening fluid, Fig. 5*A* (filled squares), is used, we observe a markedly different behavior. Varying *v* from 1 to 20 mm min^−1^, Fig. 5*C* [dark (purple) to light (green)], we find that $F(h_{0})$ is Newtonian-like, with $F/\sqrt{v}$ collapsing the data (cf. Fig. 4*C*). This is consistent with the almost-constant viscosity of this fluid at low shear rates: *η* decreases from 3 to 1 Pa s as $\dot{\gamma }$ increases from $10^{-1}$ to $10^{2}\;{\text{s}}^{-1}$. A different behavior is seen when v≥ 50 mm min^−1^, Fig. 5*C* (light lines): $F/\sqrt{v}$ no longer collapses the data, and the $h_{0}$ dependence becomes stronger, although the small-$h_{0}$ limit could not be accessed in these high *v* experiments due to load cell limits. The shear rate at the onset of this change can be estimated by using Eq. **5** for $w_{\mathrm{eff}}$ with η=1Pas, so that $\dot{\gamma }=6vw_{\mathrm{eff}}/h_{0}^{2}∼$ 160 s^−1^ at v= 50 mm min^−1^ and $h_{0}=$ 0.6 mm. This is consistent with the shear rate at which we observe shear thickening in our fluid, Fig. 5*A* (filled squares), once again supporting the validity of our analysis in terms of an effective flat plate of width $w_{\mathrm{eff}}$, and an effective viscosity set by the edge shear rate, $\eta_{\mathrm{eff}}=\eta \left({\dot{\gamma }}_{w}\right)$.

### Energy Scaling.

The speeds at which we have performed our experiments to validate our scaling analysis are far too low for realistic impact protection at $v≳1\text{m}\;{\text{s}}^{-1}$. Nevertheless, our analysis, now substantially validated by experiments, allows some predictions for higher speeds via energy scaling.

The kinetic energy scales as $v^{2}$, but *F* (and energy absorbed) scales as $v^{0.2}$ for the optimal-protection shear thinning fluid with n=0.4, Eq. **6**. For a laminate with given ($h_{i}$, *B*), the consistency *K* required for energy absorption increases with *v*. In a constant-*v* approximation,

[10] $$\begin{array}{c} \int_{0}^{h_{i}}dh_{0}\;\frac{F}{L}∼\frac{n+5}{4(1-n)}h_{i}^{\frac{4(1-n)}{n+5}}\sqrt{\frac{\mathrm{vKB}}{L}}{\left(\frac{v^{5}B}{\mathrm{KL}}\right)}^{\frac{n-1}{2(n+5)}}. \end{array}$$

So, for energy ∼ 0.25 J (e.g., m=50g for L=25mm and $v=3\;\text{m}\;\;{\text{s}}^{-1}$), our model laminate (B/L=0.18Nm, $h_{i}=1\;\;\text{mm}$) requires $K∼10^{4}$ Pa s^0.4^. For this fluid, even a low $\dot{\gamma }∼1\;\;{\text{s}}^{-1}$ would generate stresses $∼10^{4}$ Pa.

Under such conditions, our fumed silica suspensions may become brittle (22), rendering manufacturing challenging, and post-impact “self healing” may not be possible. A fluid with more complex rheology, e.g., one that thins only at the high $\dot{\gamma }$ of impact, may be more suitable. This reduces stresses at slow deformation, facilitating manufacturing, self-healing, and, perhaps, even enabling fully flexible laminates. Such rheology could be achieved using suspensions that thin after thickening, due to asperity compression (23) or a brush-like coating (24), or a polymer solution with a low-shear plateau (25). Our approach also provides insight into the mechanism of these flows and how to optimize them, for example, in forming laminate structures with unset polymer adhesives or foams, where ensuring $w_{\mathrm{eff}}\gg W$ is required for squeezing a uniform layer.

## Conclusions

Inspired by the use of shear-thickening fluids in body armors, we have established a general scaling framework for analyzing the impact response of solid-fluid laminates, which captures interactions through an effective rigid plate squeeze flow with width $w_{\mathrm{eff}}$, which scales only weakly with all parameters, Eq. **5**. Insight can, therefore, be gained by thinking in terms of a simple rigid plate squeeze flow. Strikingly, we conclude that, not thickening, but shear thinning with $\eta \propto {\dot{\gamma }}^{-0.6}$ optimizes protection, Fig. 5*D*. This arises from reducing the $F(h_{0})$ divergence, with a low $\eta_{\mathrm{eff}}$ at small $h_{0}$ (high $\dot{\gamma }$), while still absorbing the impact energy with a high $\eta_{\mathrm{eff}}$ at large $h_{0}$ (smaller $\dot{\gamma }$). These scaling predictions were substantially verified in controlled-velocity impact tests where we measured $F(h_{0})$ and imaged the pressure distribution using photoelasticity. Together, these results establish the effective rigid plate squeeze flow approximation as a useful tool for analyzing fluid–solid interactions in composites incorporating non-Newtonian fluids, with optimization shown for where the upper layer must also be protected.

Further work including flow perpendicular to *x* and *y* (26) or curvature (21), as well as normal stress differences (27), strain-dependence (28) and extensional viscosities (29), could allow predictive design of optimized fluids for realistic impact velocities. These insights could also be applicable to sports equipment (30), combining rigidification of fabrics using shear-thickening fluids from body armor (1) with squeeze flow damping using shear-thinning fluids. More generally, our scaling approach may also apply to non-Newtonian fluid–solid interaction problems arising from rubbing skin ointments (31) or eating chocolate (32) by replacing the bending equation for a thin sheet, used to calculate $w_{\mathrm{eff}}$, with the elastic, Hertzian contact deformation of a curved surface modeling the finger or tongue.

## Materials and Methods

Non-Newtonian fluids were prepared from fumed silica in poly-ethylene glycol (PEG 200, Sigma Aldrich), with a shear-thinning suspension from 7 wt% hydrophobic hexamethyldisilazane-modified Aerosil® R812S and a shear-thickening suspension from 20 wt% hydrophilic HDK® N20. Particles are ∼100 nm radius (*SI Appendix*, Fig. S3) fractal-like aggregates (33) of ≈ 3 nm primary particles. Powders were dispersed via vortex mixing, then repeated stirring and centrifugation to break agglomerates (34), similar to conching (35).

Rotational rheometry (NETZSCH Kinexus Ultra+) was performed at T= 20°C. For the shear-thickening fluid, controlled-stress measurements were made with roughened parallel plates (radius, R=10mm and gap, H=200μm); we report the rim shear rate, $\dot{\gamma }=\Omega R/H$, from the measured rotation rate and the viscosity based on the apparent stress, $\sigma =2T/\pi R^{3}$, from the applied torque, Fig. 5*A* (blue squares). Stress was applied from 1 Pa logarithmically at 10 pts/decade with 10 s equilibration and 10 s measurement at each point up to the fracture stress (3 to 10 kPa), ensuring reversibility in separate tests. For the shear-thinning fluid, rate-controlled measurements were made in a smooth cone-plate geometry (angle, α= 1° angle; R=20mm) with $\dot{\gamma }=\Omega /\sin\left(\alpha \right)$ and $\sigma =3T/2\pi R^{3}$, Fig. 5*A* (orange circles). Shear rates were applied at 5 pts/decade from $\dot{\gamma }=0.01{\text{s}}^{-1}$ to inertial ejection, $\dot{\gamma }=4,000\;{\text{s}}^{-1}$. For glycerol (99 wt%, Fisher Scientific), measurements were made at 10 pts/decade from 1 s^−1^ to 1,000 s^−1^, 5 s equilibration and 10 s measurement.

Viscosities are shown relative to Newtonian glycerol (Fig. 5*A* gray triangles, η= 1.24 Pa s). Hydrophilic silica initially weakly shear thins, before reaching a critical rate, ${\dot{\gamma }}_{c}≃100\;\;{\text{s}}^{-1}$, where further stress does not increase the rate, discontinuous shear thickening (36). This is consistent with previous results (37), with the onset of thickening occurring when the stabilizing force, attributed to the absorption of PEG onto the silica surface, is overcome and the particles enter frictional contact (38). Compared to monodisperse spheres, discontinuous shear thickening occurs at a low volume fraction, ≈11%, which may be attributed to the fractal-like nature of the particles with additional rolling constraints (39, 40).

Hydrophobic silane surface modification creates a strongly shear-thinning material (41), Fig. 5*A* (orange circles), similar to removing adsorbed surfactants (42). At low $\dot{\gamma }$ slip is observed (43), above this shear thinning with n≈0.4 (dashed line, K=38 Pa s^0.4^) occurs up to sample fracture. Around $\dot{\gamma }=100\;{\text{s}}^{-1}$, *η* for all fluids are comparable, at the range of $\dot{\gamma }$ for low-velocity impact testing. The three fluids, with comparable absolute *η* but different $\dot{\gamma }$ dependence, allow isolation of the role of fluid rheology.

Our quasi-2D controlled-velocity impact apparatus is based on a universal testing machine (Lloyd Instruments LS5, AMETEK). The force–displacement response (20 or 100 N load cell, 1 kHz sampling) is measured with $v=0.5\;\text{mm}\;{\text{min}}^{-1}\text{to}\;200\;\text{mm}\;{\text{min}}^{-1}$. Combined with a dark-field circular polariscope (FL200, G.U.N.T. Gerätebau GmbH) and a photoelastic base, qualitative pressure measurements can be made.

Our top flexible plate, Fig. 4*A*, was 25mm×75mm×0.3mm glass. The base was a 10 mm-thick piece of cut silicone elastomer [Sylgard 184, Dow Chemical Company, 5:1 cross-linker ratio, degassed and cured at 25°C for 48 h, E= 1.5 MPa (44)]. The silicone becomes birefringent under applied loads, generating photoelastic contrast as the polymer chains stretch and align with strain (45). The constraining panels were sealed with silicone oil (10,000 cSt, Sigma Aldrich). For non-Newtonian fluid force–displacement tests, glass was on top of the base (compliance, $k=80\;\;\text{N}\;{\text{m}}^{-1}$, $h_{i}=0.76\;\;\text{mm}$); otherwise $k=50\;\;\text{N}\;{\text{mm}}^{-1}$, $h_{i}=0.7\;\text{mm}$.

For force–displacement measurements, the initial gap, $h_{i}$, and zero displacement, Δx=0, were set with no fluid. After loading the fluid, the laminate was allowed to come to equilibrium, F=0 and Δx=0. The impactor was then moved down 0.8 mm at a fixed speed, *v*, recording F(t) and Δx(t) from which F(Δx) was reconstructed. The gap, $h_{0}=h_{i}-\Delta x+F/k$.

To infer the fluid pressure, we used a polariscope to probe stress in the base, giving finer spatial resolution than transducer arrays (46, 47). Stress-induced intensity patterns in the PDMS, I(x,y,t), were recorded using a camera (Nikon Z6, 3,840×2,160 30 Hz, 8-bit grayscale). Instead of precisely quantifying the stress (48), we sought to establish the extent of any high-pressure region. A narrow region at the top of the base layer is isolated in recording, 700 × 10 px^2^, Fig. 4*A* (red outline). The change in intensity from the quiescent state at the start of recorded movies, ΔI(x,y,t), is averaged vertically, ΔI(y,t), and smoothed on short-length scales using a Savitzky–Golay filter. The intensity is normalized to saturation (ISO 1200 and shutter speed 1/125).

## Supplementary Material

Appendix 01 (PDF)

## Data Availability

Data are available in Edinburgh DataShare at https://doi.org/10.7488/ds/7556 (49).

## Footnotes

^*^Initial contact is not accurately described, but for large deformations (*h*_0_ → 0) this can be neglected.

^†^Larger *w*_eff_ increases *Q* and $\partial_{y}p\propto w_{\mathrm{eff}}$, such that $p\propto w_{\mathrm{eff}}^{2}$ and $F\propto w_{\mathrm{eff}}^{3}$. The bending moment in the plate $\propto w_{\mathrm{eff}}^{4}$, the angular deflection $\propto w_{\mathrm{eff}}^{5}$ and, ultimately, $\Delta h\propto w_{\mathrm{eff}}^{6}$.
