# Supplementary material for: Optimizing non-Newtonian fluids for impact protection of laminates
Source: Proc Natl Acad Sci U S A. 2024 Feb 27;121(10):e2317832121. doi: 10.1073/pnas.2317832121 (PMC10927517; doi:10.1073/pnas.2317832121)
Supplement: Supplementary file 1 — Appendix 01 (PDF) [file pnas.2317832121.sapp.pdf]

Supporting Information for

Optimising non-Newtonian fluids for impact protection of laminates

James A. Richards, Daniel J. M. Hodgson, Rory E. O'Neill, Michael E. DeRosa and Wilson C. K. Poon

James A Richards.
E-mail: james.a.richards@ed.ac.uk

This PDF file includes:

Supporting text
Figs. S1 to S3
SI References

Supporting Information Text

Effect of curvature

In the main text, we define an effective width w_{eff} to denote the region in which there are significant pressure gradients. Since $\partial_y P \sim h_0^{-3}$, Eq. (2), this region is delimited by the criterion of $\Delta h \lesssim h_0$. Over the same region, we have claimed that it is reasonable to approximate the top plate as flat, because it is only weakly curved. Here, we demonstrate the truth of this claim via calculating the shear rate. The same calculation will be relevant when we consider non-Newtonian fluids.

For a rigid curved plate, the gap can be approximated by $h(y) = h_0 + y^2/2\rho$, for a radius of curvature, ρ. With $Q = vy$ (unchanged from a flat plate) by conservation of volume, the wall shear rate is

$$\dot{\gamma} = \frac{6Q}{h^2} = \frac{6vy}{\left(h_0 + \frac{y^2}{2\rho}\right)^2}. \qquad \text{[S1]}$$

Normalising lengths by ρ and plotting the shear rate with y, Fig. S1, demonstrates that the shear rate for small y is equivalent to a flat plate, *cf.* solid and dotted lines. Here $h \approx h_0$ and $\dot{\gamma} \propto vy/h_0^2$. At large y, the growth in h dominates and the shear rate drops, $\dot{\gamma} \propto \rho^2 v/y^3$. For narrower gaps, or more curved surfaces $h_0/\rho \ll 1$, the flat-plate–like region where shear rate is $\propto y$ becomes smaller (dark to light lines). The transition defined by $\max(\dot{\gamma})$, occurs at $y = \sqrt{2h_0\rho/3}$, Fig. S1 (dotted line). At this point the gap has increased by $h_0/3$. Within a numerical constant of order unity, this is the same criterion as that we have used to define w_{eff} ($\Delta h \sim h_0$) based on Eq. (2) alone and used in our scaling analysis. The shear rate in the curved case is $9/16 \approx 0.56$ times that for a plate, while the pressure gradient is ≈ 0.42 that for a flat plate.

Derivation of closure relation for non-Newtonian fluids

To describe the non-Newtonian response of the fluid we substitute w_{eff}, Eq. (5), into $\dot{\gamma}_w \simeq 6vw_{\text{eff}}/h_0^2$ to obtain

$$\dot{\gamma}_w \simeq 6\left(\frac{Bv^5}{12\eta h_0^8 L}\right)^{1/6}. \qquad \text{[S2]}$$

Substituting $\eta = K\dot{\gamma}^{n-1}$ and solving for $\dot{\gamma}_w$, we then find

$$\dot{\gamma}_w \simeq 6^{\frac{6}{n+5}}\left(\frac{Bv^5}{12Kh_0^8 L}\right)^{\frac{1}{n+5}}, \qquad \text{[S3]}$$

so that the effective Newtonian viscosity is

$$\eta_{\text{eff}} = K\dot{\gamma}_w^{n-1} \simeq 6^{\frac{6(n-1)}{n+5}} K\left(\frac{Bv^5}{12Kh_0^8 L}\right)^{\frac{n-1}{n+5}} \qquad \text{[S4]}$$

As in our reduced Newtonian FSI solution, η_{eff} sets the effective width of the squeeze flow region. From Eq. (5), we then find

$$w_{\text{eff}} \simeq \left(\frac{Bh_0^4}{12\eta_{\text{eff}} vL}\right)^{\frac{1}{6}} \simeq 6^{\frac{1-n}{n+5}}\left(\frac{Bh^4}{12KvL}\right)^{\frac{1}{6}}\left(\frac{Bv^5}{12Kh_0^8 L}\right)^{\frac{1-n}{6(n+5)}}. \qquad \text{[S5]}$$

This sets the force that stops the impact and bends the glass. Again, from Eq. (5), we find

$$\frac{F}{L} \simeq \frac{12\eta_{\text{eff}} v w_{\text{eff}}^3}{h_0^3} \simeq \frac{12\eta_{\text{eff}} v}{h_0^3}\left(\frac{Bh_0^4}{12\eta_{\text{eff}} vL}\right)^{\frac{1}{2}} = \frac{\sqrt{12}}{h_0}\left(\frac{vB}{L}\right)^{\frac{1}{2}}\eta_{\text{eff}}^{\frac{1}{2}}, \qquad \text{[S6]}$$

from which,

$$\frac{F}{L} \simeq \frac{\sqrt{12}\times 6^{\frac{3(n-1)}{n+5}}}{h_0}\left(\frac{KvB}{L}\right)^{\frac{1}{2}}\left(\frac{Bv^5}{12Kh_0^8 L}\right)^{\frac{n-1}{2(n+5)}}. \qquad \text{[S7]}$$

Again, we recover the Newtonian case if $n = 1$, $K = \eta$.

Particle sizing

Throughout, we have treated our experimental non-Newtonian fluids as continua. This requires that the minimum gap size we resolve during impact, $\approx 50\,\mu\text{m}$ (Fig. 4C), always remains significantly larger than the size of the particles and agglomerates in our suspensions. We have therefore measured the particle size distribution (PSD) of our experimental suspensions to ensure proper dispersal before use.

Dilute suspensions ($\varphi = 0.05\,\text{wt}\%$) were prepared in a compatible solvent, distilled water (N20) or ethanol (R812S). The PSD was then determined using dynamic light scattering (DLS, ALV LSE-5004 at 632.8 nm and 45° scattering angle) using a regularised fit of the decorrelation function, Fig. S3 [N20, light (blue) lines and R812S, dark (orange) lines]. This is presented unweighted as fumed silica is a fractal-like particle and the standard radius dependence does not apply (1). Fumed silica

was dispersed via three methods. Firstly via simple vortex mixing that leaves agglomerates intact,(2) solid lines. Secondly, when dispersed with further high energy input sonication (Sonics vibra-cell, 500 W power with a tapered probe at 20% power with 10 s pulses for 2 min), dashed line. Finally, samples as prepared for laminate testing were diluted and vortex mixed to $\varphi = 0.05$ wt%, dotted lines.

For particles mixed using dilute shear, the PSD span is broad, being spread from 150 nm upwards, peaking at 220 nm for R812S and 410 nm for N20, then with a tail of large radius particles up to 0.5 µm or 2.5 µm respectively, Fig. S3 (solid lines). When particles are dispersed via high-energy probe ultrasound, a narrower PSD is found with peaks at 100 nm and 150 nm (dashed lines) associated with the diameter of the permanently fused aggregates of primary particles. Comparable peaks are found with high volume fraction shear followed by dilution, dotted lines. This indicates that the mixing method used for suspension preparation breaks up agglomerates.

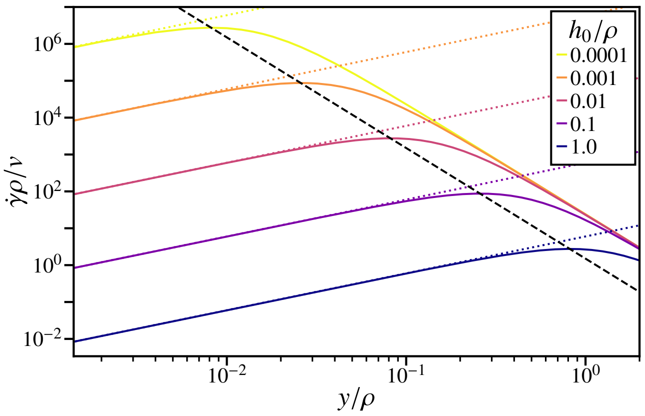

Fig. S1. Impact of curvature on wall shear rate. Shear rate normalised by velocity and radius of curvature, $\dot{\gamma}v/\rho$, as a function of normalised distance from point of impact, y/ρ. Lines: solid, shear rate for narrowing gap (dark to light, see inset legend for h_0/ρ values); dotted, shear rate for flat plate, $h = h_0$; and, dashed, $y/\rho = \sqrt{2h_0/3\rho}$, defining maximum shear rate and scale for transition from high pressure region at $\Delta h = y^2/2\rho = h_0/3$.

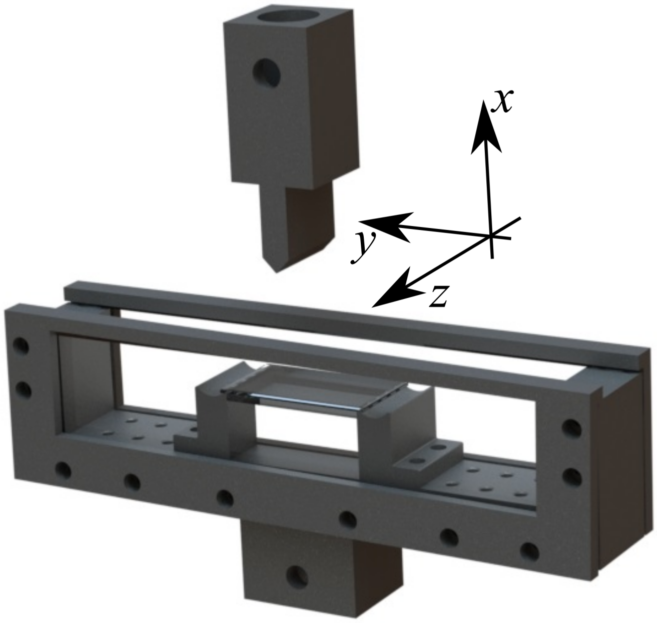

Fig. S2. 3D rendering of quasi-2D laminate geometry and impactor showing movable support points for flexible glass layer and confining panels to prevent flow along z direction.

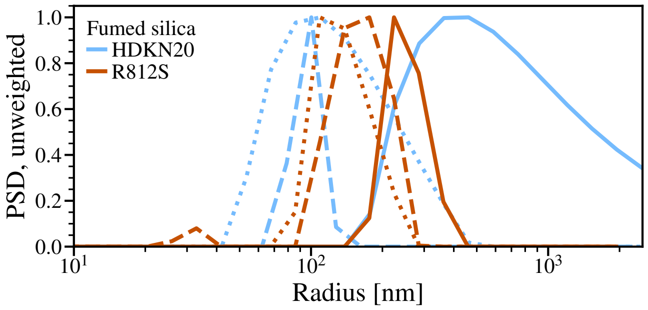

Fig. S3. Particle size distribution (PSD) from dynamic light scattering for hydrophilic (HDK N20, blue) and hydrophobic (R812S, red) fumed silica for various dispersal methods (lines: solid, dilute shear; dashed, sonicated; and dotted, concentrated shear and dilution). Particle size distribution normalised to peak value, presented as unweighted results from regularised exponential analysis.

References

1. N Ibaseta, B Biscans, Fractal dimension of fumed silica: Comparison of light scattering and electron microscope methods. *Powder Technol.* **203**, 206–210 (2010).
2. SW Kamaly, AC Tarleton, NG Özcan-Taşkın, Dispersion of clusters of nanoscale silica particles using batch rotor-stators. *Adv. Powder Technol.* **28**, 2357–2365 (2017).
